# Alerting relatives about heritable risks: the limits of confidentiality

**DOI:** 10.1136/bmj.k1409

**Published:** 2018-04-05

**Authors:** Anneke Lucassen, Roy Gilbar

**Affiliations:** 1 Faculty of Medicine, University of Southampton, Wessex Clinical Genetics Service annekel@soton.ac.uk; 2 Netanya Academic College, Netanya, Israel; 3 School of Law, University of Leicester, Leicester

## Abstract

Are there situations when sharing a patient’s genetic information with relatives without consent is acceptable? **Anneke Lucassen** and **Roy Gilbar** discuss

John has recently received a diagnosis of Huntington’s disease. This serious hereditary condition has no cure and early death (aged 40-60) is likely. An affected person has a 50% chance of passing the condition on to their children. Those who inherit the causative mutation (a triplet repeat or “stutter” in the DNA) are likely to develop the disease in mid-life, or earlier if the stutter has enlarged, which can sometimes happen if it is inherited paternally. John’s daughter, Clare, knows her father is ill but not that his condition is heritable. She is pregnant and mentions this to John’s clinicians. They wonder whether she has a right to know that he has a heritable condition, but John worries about Clare and that she might terminate her pregnancy. He therefore refuses consent for Clare to be told.

This scenario, recently discussed by English courts in ABC *v* St George’s Healthcare NHS Trust and others (box 1),^1^ raises questions that clinicians increasingly face—namely, whether, and how, genetic information discovered in one patient might be communicated to relatives at risk of inheriting it. Tensions around confidentiality and responsibilities to others are well described in practices around, for example, sexually transmitted diseases (STDs), yet for heritable risks the prevailing discourse remains one that gives prominence to patient confidentiality. This is presumably because, until recently, few treatments were available to prevent or ameliorate the course of genetic conditions.

Box 1 **the ABC case**

ABC is the first case in English law to deal with non-disclosure of a patient’s genetic information to a relative.^22^ ^23^ ^24^ Similar to our scenario of John and Clare, in ABC, a daughter claimed that her father’s clinicians were negligent for not telling her that he had Huntington’s disease. The claim was initially struck out by the High Court as it was held that there could be no duty of care to relatives in such circumstances.^25^ ^26^

The Court of Appeal reversed this decision in May 2017, holding that, depending on the circumstances of a case, it may be fair, just, and reasonable to impose a legal duty on a clinician to inform a patient’s relatives about their increased genetic risk.

The case can now go to trial, where the court would examine whether the treating clinicians met the reasonable professional standard when respecting a patient’s refusal to disclose his genetic status to his daughter. The Appeal Court emphasised that relatives’ interests in autonomy and disclosure are no less important than a patient’s rights to autonomy and confidentiality and that a clinician’s duty towards relatives “would serve to ensure that a proper balancing exercise is performed by the clinician.” The court held that the law in this area should reflect the professional obligations clinicians already hold towards relatives.^1^

Now that surveillance and interventions are available for people who know they are at risk of certain conditions, we may need to consider contact tracing of relatives more like we do for STDs. Furthermore, genetic technologies have become much cheaper and quicker, and genetic or genomic testing is now entering mainstream medical practice^2^ as well as being more available direct to the consumer commercially. Knowing if, or when, it might be appropriate to alert others about risks discovered through the testing of one person therefore becomes an issue for all doctors.

## Confidentiality and its limits

General Medical Council (GMC) guidance emphasises that confidentiality is usually the rule but that disclosure to others might be an exception.^3^ Communication of information given in confidence is generally permitted only if the patient consents or if each of three criteria are met: the patient refuses to inform others, an identifiable person (relative) is at serious risk of harm, and such harm might be prevented by disclosure.^4^

In STDs, disclosure might be justified to prevent transmission of a disease. In genetics, the harm prevention is the access to surveillance or treatments that a relative would otherwise not know to access. In familial polyposis, for example, relatives live on average 20 years longer if they have regular bowel surveillance and preventive surgery. In familial breast or ovarian cancer, women can access early screening, chemoprevention, or risk reducing surgery only if they know they have inherited the BRCA1/2 mutation. Avoidance or termination of pregnancy (to prevent transmission of a condition such as Huntington’s disease) might also be considered harm prevention.

Hence, according to the GMC guidance, John’s doctors should have considered whether his refusal to allow Clare to be told about her heritable risks could be over-ridden. If their conclusion was that informing Clare would help her avoid serious harm, their disclosure would have been justified despite John’s refusal. This balancing exercise is not easy, but it is important to remember that John had no absolute right to veto disclosure.

## What type of information is confidential?

The BMA states that identifiable patient information, “whether written, computerised, visually or audio recorded, or simply held in the memory of health professionals is subject to the duty of confidentiality.”^5^ This includes clinical information about diagnosis or treatment, photos or other images of the patient, and the details of the doctor and the clinics the patient attends. 

The Data Protection Act 1998 defines personal data as data that “relate to a living individual who can be identified by those data,” or by those data together with other information held by the data controller (eg, a clinician). The act considers health records as “sensitive personal data” that can be disclosed only if certain conditions are met (such as patient consent).^6^ The forthcoming European General Data Protection Regulation defines personal data as “any information relating to an identified or identifiable person,” and perceives genetic information as sensitive personal data.^7^

However, since 99.9% of the genetic code is the same in all humans this cannot be considered sensitive, just as it cannot be considered sensitive to say that a person has lungs or kidneys. It is the (0.1%) differences in genetic codes that lead to potential sensitivity. Biological relatives share an even greater proportion of their genetic code, so a particular BRCA1 mutation, for example, may not identify an individual but rather a group of related individuals who have a family history of breast and ovarian cancer. Alerting relatives that their family history may mean they have an increased chance of developing a condition therefore does not breach confidentiality because no identifiable information is communicated in such a statement, even if genetic findings in one person first led to that conclusion.

We have previously argued that relatives’ interests in the patient’s genetic test results should be taken into account and that disclosure practices should be amended accordingly.^8^ ^9^ This view has been endorsed in clinical practice guidance^10^ but has not yet gained wide enough acceptance by clinicians to be applied in John and Clare’s case.^11^ Most Western countries seem to take a similar position to the UK, presumably because clinical practice usually prioritises the consent and confidentiality of the patient under investigation. As genetic and genomic medicine is becoming more integral to general medical practice we need to have a debate about when relatives’ interests play a part in decisions.^12^

## Family history and confidentiality

In our scenario, John is known to have a family history of dementia, and some of his relatives had the movement disorder characteristic of Huntington’s disease before they died. Clare remembers John’s mother having dementia at an early age. John’s clinicians used this information to consider a diagnosis of Huntington’s in John. Clare observes John’s symptoms and asks his clinicians whether they have implications for her. John’s clinicians are now in a difficult position. They need to place a high value on keeping his clinical information confidential, but can they tell Clare her family history may have important consequences for her without breaching John’s confidentiality?

We suggest that in these situations clinicians could say to relatives like Clare, “Your family history suggests there might be an inherited predisposition in your family,” because John has refused disclosure of his information not of information that is not unique to him. Clare could then seek further advice about whether testing might be available, without any breach of John’s confidence. Clare’s test results might eventually lead her to infer things about John, but we would argue that this is not the same as a breach of his confidence because the information discovered is not about a particular individual. If Clare concludes that her father has the same genetic finding, it does not reveal confidential information about John that she is not already party to.^13^ Since family members share the same gene pool, certain inferences about their genetic status will be inevitable regardless of clinicians’ disclosure.

In some cases it will not be possible to use a family history to suggest to a relative they are at risk: clinical findings and their heritable explanation may be evident in only one person, for example. Even here we argue, alerting relatives to their risk is not the same as breaching confidentiality, because it is not unique information that is being disclosed.

We do not underestimate the difficulties of implementing such an approach systematically. The threshold of risk, severity of the condition, as well as the interventions that would be available after disclosure, would need to be defined. Furthermore, there are practical difficulties in communicating with people who might not be easy to find, or who might not want to receive such information. For now, we simply posit that relatives can sometimes be told about their genetic risks without breaching the confidences of the patient in whom the heritable tendency was first identified. This does not equate to a duty to contact all, but to a responsibility to contact some people, some of the time.

A next step therefore might be to agree that clinicians could consider disclosure in cases such as that of John and Clare. Documenting these considerations and how decisions about disclosure are made will be an important part of this process. We then need a much wider debate about when, and at what level of risk or available preventive options, such disclosure might be more proactive, and how.

## Empirical evidence about familial communication

Families often share their genetic information, and it is rare for a patient to explicitly refuse to inform relatives.^14^ However, this does not mean that appropriate communication always takes place. Patients may find such communication difficult for many reasons, including lack of contact, not finding an appropriate time, or not understanding its importance.^15^ Many genetic services offer “family letters” for patients to pass on to relatives, but these are also not always effective.^16^

Other research suggests that both patients and clinicians within genetic services already distinguish between personal and familial information on some level and have no objection to disseminating familial genetic information when the benefit from disclosure is high in terms of surveillance or treatment.^17^ Patients think that their relatives should have the opportunity to receive relevant familial genetic information and perceive reproductive choices in the same way as they do treatment options.^18^

## Professional guidelines and the law

In the UK, the GMC considers disclosure to others without consent is justified “if failure to do so may expose others to a risk of death or serious harm.”^3^ For genetic information, the GMC says that if a patient refuses to disclose relevant information to relatives, clinicians need to balance their duty to make the care of their patient their first concern against their duty to help protect another person from serious harm. The GMC suggests that doctors should not reveal the patient’s identity when contacting relatives, thus acknowledging that familial and individual information might be separable.^3^

English law, like professional guidelines, imposes a duty of confidentiality on clinicians and grants discretion to warn relatives.^19^ Whereas the GMC can initiate disciplinary actions against doctors who transgress guidelines, the courts can direct clinicians to pay compensation to patients for breaching confidentiality.

The GMC if approached by Clare might conclude that John’s clinicians failed to adequately consider GMC guidance on confidentiality. However, if Clare wants compensation, the courts first need to examine whether her father’s clinicians owed her a duty of care.^1^ If they did, the courts must decide if clinicians breached this duty of care and whether it caused the harm the claimant experienced.

The assessment of whether clinicians owe a duty of care (to inform, in this context) to particular relatives needs to meet three criteria: Could they have foreseen that their (in)action might cause harm? Could they have identified a particular relative as being at risk? And is it “fair, just, and reasonable” to impose such a duty?^20^ ^21^

## Implications for clinical practice

In the ABC case (box 1) the Court of Appeal’s decision that doctors may have a legal duty to inform a patient’s relatives of their risk of Huntington’s disease has important implications. Although the court held that such a duty of care to relatives might be limited to genetic practices, this limitation will reduce as genetics becomes part of every branch of medicine. Clinicians treating patients whose conditions have a major, and definable, inherited component (for example, a BRCA1 or 2 mutation) need to consider not only their patients’ wishes and interests but also those of relatives at risk, who may in turn become patients to be advised about surveillance or risk reducing measures.

The ABC case therefore guides clinicians to use their professional judgment and consider whether harm might be prevented by disclosure of (potentially) familial rather than personal genetic information. What will now need to be determined in cases where patients explicitly refuse disclosure to relatives, is how to fulfil this duty—namely, if, how, when, and by whom might Clare be informed, and which other relatives such a duty also applies to. In practice, we suggest that clinicians call on multidisciplinary expertise, including regional genetic or genomic services, clinical ethics committees, and professional guidance from the GMC and royal colleges.^3^ ^10^

**Key messages**

Increasing adoption of genetic technology throughout medicine means that questions about communication of heritable risks to family members will arise more often
Clinicians often believe that preservation of confidentiality prevents them from disclosing genetic information to relatives at risk without the patient’s explicit consent
A recent UK court case highlights that clinicians need to weigh the potential harms of disclosure against the potential benefits
We argue that in many cases appropriate relatives can be alerted without a breach of confidence of clinical information

## Notes

Contributors and sources: The authors contributed equally to the article. It arose out of a series of discussions about the topic at clinical and academic meetings. We acknowledge discussions with many parties but in particular those with Michael Parker of the Ethox Centre, University of Oxford and Genethics UK. AL is a professor in clinical genetics at the University of Southampton. She chairs her hospital’s clinical ethics committee and helps run Genethics UK, a national forum for discussion of difficult ethical and legal issues arising in genetic practice. She has published on the familial aspects of genetic information and its communication to those to whom it is of potential interest. RG’s research examines the legal and bioethical aspects of genetic practice. He has published on legal and bioethical aspects of confidentiality and disclosure of medical information to family members. AL’s work is supported by funding from a Wellcome Trust collaborative award 208053/Z/17/Z.

Provenance and peer review: Not commissioned; externally peer reviewed
